# Piezoelectric Materials and Sensors for Structural Health Monitoring: Fundamental Aspects, Current Status, and Future Perspectives

**DOI:** 10.3390/s23010543

**Published:** 2023-01-03

**Authors:** Min Ju, Zhongshang Dou, Jia-Wang Li, Xuting Qiu, Binglin Shen, Dawei Zhang, Fang-Zhou Yao, Wen Gong, Ke Wang

**Affiliations:** 1 Research Center for Advanced Functional Ceramics, Wuzhen Laboratory, Jiaxing 314500, China; 2 Center of Advanced Ceramic Materials and Devices, Yangtze Delta Region Institute of Tsinghua University, Jiaxing 314500, China; 3 State Key Laboratory of New Ceramics and Fine Processing, School of Materials Science and Engineering, Tsinghua University, Beijing 100084, China

**Keywords:** piezoelectric materials, sensors, structural health monitoring

## Abstract

Structural health monitoring technology can assess the status and integrity of structures in real time by advanced sensors, evaluate the remaining life of structure, and make the maintenance decisions on the structures. Piezoelectric materials, which can yield electrical output in response to mechanical strain/stress, are at the heart of structural health monitoring. Here, we present an overview of the recent progress in piezoelectric materials and sensors for structural health monitoring. The article commences with a brief introduction of the fundamental physical science of piezoelectric effect. Emphases are placed on the piezoelectric materials engineered by various strategies and the applications of piezoelectric sensors for structural health monitoring. Finally, challenges along with opportunities for future research and development of high-performance piezoelectric materials and sensors for structural health monitoring are highlighted.

## 1. Introduction

Structural health monitoring (SHM) is a ubiquitous technology to evaluate the status and integrity of structures, and even predict their lifetime by constantly collecting and analyzing the data acquired from the sensors integrated in the structures [1,2]. SHM is of particular importance for complex engineering structures, which require costly maintenance, to significantly lower the maintenance cost and guarantee the safety and reliability thereof [3]. It is estimated that the market of SHM reached up to USD 2 billion in 2022 [4]. In addition, driven by the growing demands of internet of things, the SHM market is predicted to expand at a high compound annual growth rate of 14.6 in the following 5 years.

Sensors with high sensitivity, good reliability, and low cost are the cornerstone for the structural health monitoring (SHM). Various kinds of sensors have been developed to realize the SHM, such as strain gages [5], accelerometers [6], fiber optical sensors [7,8], displacement sensors [9], piezoelectric sensors [10], and laser Doppler vibrometers [11]. Piezoelectric materials are capable of becoming electrically polarized upon the application of external stress or deform in response to electrical stimuli. Therefore, sensors based on piezoelectric effect could be used as multipurpose sensors to realize the SHM using a variety of methods, including electromechanical impedance technology [12,13], ultrasonic propagation monitoring [14], acoustic emission [15], and stress monitoring [16]. Compared with other monitoring sensors or techniques, piezoelectric sensors have numerous advantages, such as small size, light weight, low cost, availability in a variety of formats, high sensitivity, and so on.

To meet the growing demands for high-performance piezoelectric sensors for SHM, there has been considerable research interest in this domain. In the recent years, lots of work has been focused on the piezoelectric sensors, such as piezoelectric transducers, smart aggregates, direct deposition piezoelectric sensors on structure, flexible smart sensors, and so on [17,18,19,20,21]. Numerous review articles have been published on the applications of piezoelectric SHM, for instance, bonded structures [22], polymer-matrix composites [23,24], aircraft applications [14], wind turbine blades [25,26], bridge applications [27], or other engineering structures [10]. Moreover, some reviews were focused on the monitoring techniques, such as impedance-based SHM [12,13], ultrasonic Lamb, or both [28].

In this review, we provide an overview of the currently available piezoelectric materials and sensors for SHM. In particular, focuses are placed on the high-performance piezoelectric materials, covering organic piezoelectrics, inorganic piezoelectrics and piezoelectric composites, engineered by various strategies, and piezoelectric sensors operated in active and passive modes for SHM. We conclude by highlighting some challenges and opportunities for future developments.

## 2. Piezoelectric Effect

**Figure 1:**
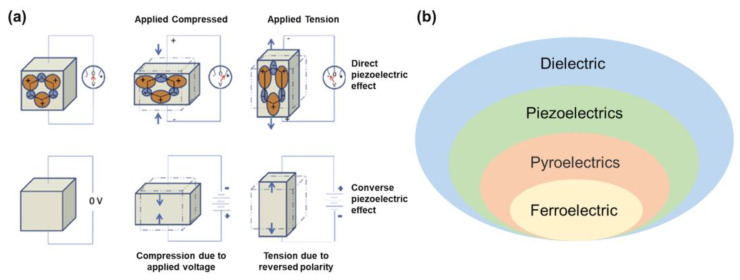
(**a**) Schematic of direct and converse piezoelectric effect [30]; (**b**) the affiliations of dielectric, piezoelectric, pyroelectric, and ferroelectric.

The working principle of a piezoelectric sensor depends on “piezoelectric effect” of piezoelectric materials, first discovered by the Curie brothers in 1880 [29]. They found that, when an external force (pressure or tension) is applied in a specific direction of some dielectric crystals, the surface of both ends of the crystal will generate positive and negative bound charges of equal amount of electricity, and the density of bound charges is proportional to the magnitude of the applied stress, which is called the “positive piezoelectric effect”. Subsequently, G. Lippman and the Curie brothers predicted and confirmed the existence of inverse piezoelectric effect in theory and experiment, respectively, that is, the material with piezoelectric effect will produce corresponding deformation under a certain electric field, and the deformation of the material will be restored when the applied electric field is removed. The positive piezoelectric effect and the inverse piezoelectric effect are reciprocal inverse effects, which jointly characterize the ability of piezoelectric materials to realize the conversion of mechanical energy and electric energy. The schematic diagram is shown in Figure 1a [30].

Whether the piezoelectric effect exists in a crystal depends on the symmetry of its crystal structure. Neumann principle requires that the symmetry of any macroscopic physical properties of a crystal should include the symmetry of the point group to which the crystal belongs. Therefore, it is possible for crystals without symmetry centers to have piezoelectric effects. Strict mathematical derivation shows that there are 32 kinds of macroscopic symmetries of crystals, that is, 32 kinds of point groups without translation operation. According to symmetry, these 32 point groups can be divided into two categories: central symmetry and noncentral symmetry. Of these, 11 are centrally symmetric, so only 21 are likely to have piezoelectric effects. However, although the point group 432 (O) has no symmetry center, its symmetry is very high and it does not have the piezoelectric effect. In addition, the spontaneous polarization of a part of the pyroelectric body can be reversed under the action of an applied electric field. Such crystals are called ferroelectrics. It must be pointed out that piezoelectric material must first be dielectric. Secondly, some piezoelectric materials are pyroelectric or ferroelectric. The affiliations of dielectric, piezoelectric, pyroelectric, and ferroelectric are shown in Figure 1b.

## 3. Piezoelectric Materials

### 3.1. Inorganic Piezoelectric Materials

**Figure 2:**
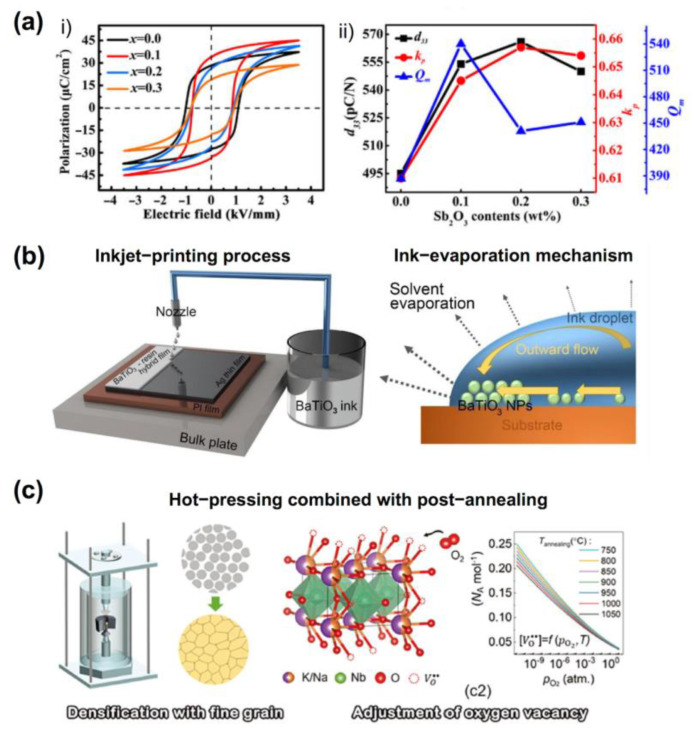
(**a**) P(E) loops of unpoled (xSb, Mn)-doped PZT ceramics at 1 Hz (i) and d_33_, k_p_, and Q_m_ of poled (xSb, Mn)-doped PZT ceramics (ii) [37]. (**b**) Schematic illustrations for a facile inkjet-printing process and ink solvent evaporation mechanism in the printed droplet [53]. (**c**) Hot-pressing combined with a post-annealing process for the KNN preparation [46].

Inorganic piezoelectric materials include piezoelectric single crystals (e.g., quartz), piezoelectric ceramics, and piezoelectric films, and their development process is the process of improving their piezoelectric properties. The piezoelectric properties of piezoelectric materials are mainly controlled by element doping. The development of an inorganic piezoelectric material system has gone through two stages: single component (such as BaTiO_3_ [31] and PbTiO_3_ [32]) and morphotropic phase boundary (such as lead zirconate titanate (PZT) [33,34], (1-x)Pb(Mg_1/3_Nb_2/3_)O_3_-xPbTiO_3_ (PMN-PT) [35], and Ba(Zr_0.2_Ti_0.8_)O_3_-x(Ba_0.7_Ca_0.3_)TiO_3_ (BCT-BZT) [36]). With the development of material systems, the properties of inorganic piezoelectric materials have been greatly improved. Among them, PZT is one of the piezoelectric materials with outstanding comprehensive performance. As shown in Figure 2a, Lin et al. prepared Sb_2_O_3_-doped PZT-based piezoelectric ceramics that possess simultaneously enhanced piezoelectric coefficient d_33_ and large mechanical quality factor Q_m_ value [37].

However, with the increasing application requirements and scenarios of piezoelectric materials, the demand for high-performance piezoelectric materials is increasingly urgent. The existing material systems developed based on traditional methods have reached a bottleneck stage, which is gradually difficult to meet the requirements of precision sensing. In recent years, studies have shown that the doping of new complex multi-component components (such as Sm-doped PMN-PT [38]) can greatly improve the piezoelectric properties of piezoelectric materials, and the piezoelectric coefficient can be more than twice that of the existing piezoelectric system. The new ultra-high performance piezoelectric materials have the characteristics of multi-rare-earth element doping and multi-scale complex structures, which make the traditional methods, such as composition traversal preparation methods and single-scale material structure characterization methods, have a huge workload. To meet the increasing demand for high-end electrical materials, the development of new ultra-high-performance piezoelectric materials and their devices based on artificial intelligence methods, such as machine learning or high-throughput computation and experiments, has become an inevitable trend in the future of the sensing field.

In addition, due to the toxicity of Pb, environmentally benign lead-free piezoelectrics have received intensive attention, such as BaTiO_3_ [39,40,41], sodium bismuth titanate (BNT) [42,43,44], and potassium sodium niobate (KNN) [45,46,47,48,49,50,51,52]. In Figure 2b, Jong et al. demonstrated a BaTiO_3_ hybrid film by adopting a simple and facile inkjet-printing process [53]. By optimization of the ceramic particle movement in the flow that occurred by solvent evaporation in a droplet of ink, they successfully formed the BaTiO_3_ ceramic layer, whose packing density of ceramic particles was over 55% in volume. Wang et al. proposed the engineering of oxygen vacancy, aiming to solve the antagonistic relationship between Q_m_ and d_33_ in hard piezoelectrics of KNN by the hot-pressing combined with a post-annealing process, leading to an enhancement of Q_m_ by 60% (Figure 2c) [46]. 

### 3.2. Organic Piezoelectric Materials

**Figure 3:**
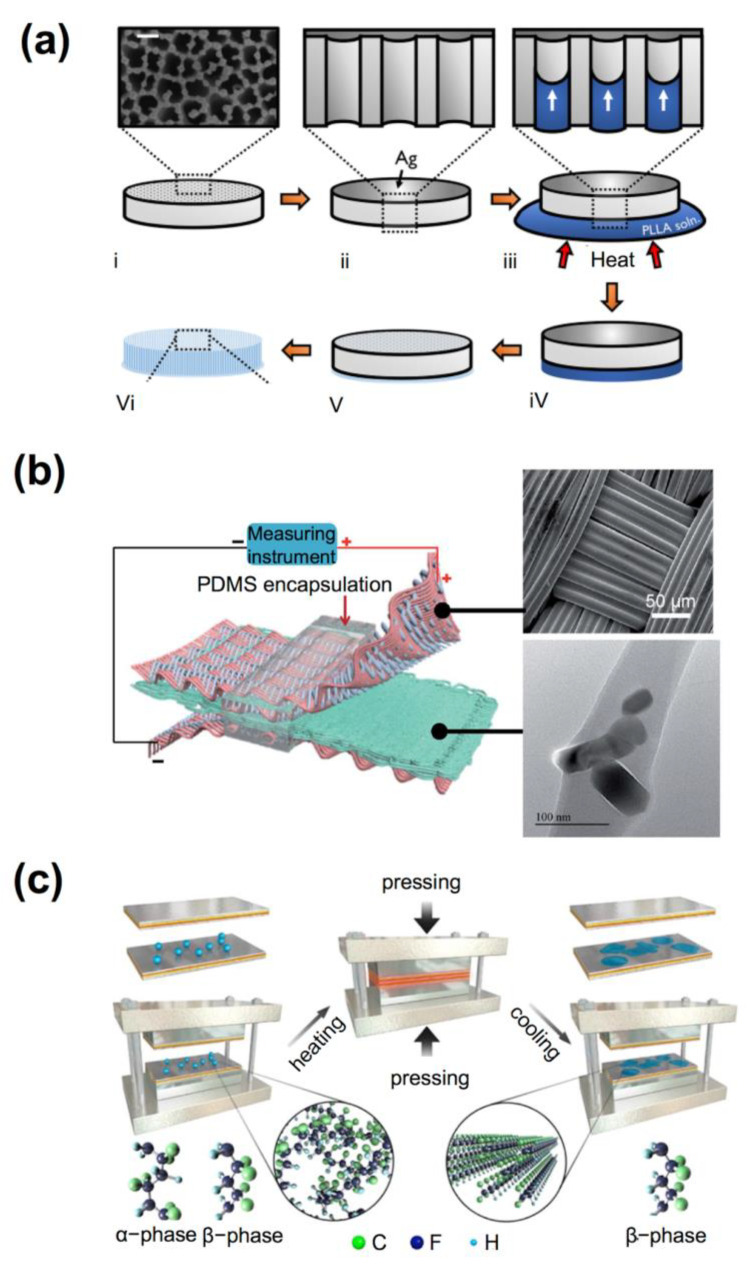
(**a**) A schematic of the temperature controlled capillary infiltration process used to grow the PLLA nanowires [59]; (**b**) schematic diagram of ZnO-nanoparticle-reinforced PVDF nanofibers by electrospinning [60]; (**c**) a typical hot-pressing method adopted to fabricate ultrathin PVDF nanoflakes [55].

Organic piezoelectric materials are mainly piezoelectric polymers, which are widely used in flexible sensors due to their good mechanical properties, including poly(L-lactide) (PLLA) [54], poly(vinylidene fluoride) (PVDF) [55], poly(vinylidene fluoride trifluoroethylene) (P(VDF-TrFE)) [56], polyimides (PI) [57], polyacrylonitrile (PAN) [58], etc. As shown in Figure 3a, Sohini et al. first reported the direct observation of shear piezoelectricity in highly crystalline and oriented PLLA nanowires grown by a novel template-wetting method [59]. 

PVDF is the most typical organic piezoelectric material, which is versatile and light in weight in comparison to piezoelectric ceramics. In consequence, thin films of any desired form can be drawn into them, giving them an advantage over piezoceramics in various applications involving complex designs of sensors. Despite organic piezoelectric materials having lower electromechanical coupling compared to inorganic counterparts, other characteristics that make the piezoelectric polymers attractive are their low electrical permittivity, low acoustic impedance, high voltage sensitivity, and relatively lower cost. Mandal et al. designed ZnO-nanoparticle-reinforced PVDF nanofibers by electrospinning (Figure 3b) [60]. Nanofibers act as the active layer and interlocked conducting microfiber composite mats as electrodes to convert the mechanical energy into electrical power. Taking advantage of high flexibility and easy processability of PVDF, Wang et al. fabricated ultrathin PVDF nanoflakes with thicknesses down to 7 nm by using a hot-pressing method (Figure 3c) [55]. This thermo-mechanical strategy simultaneously induces robust thermodynamic α to electroactive β phase transformation, with β fraction as high as 92.8% in sub-10 nm flakes.

### 3.3. Composite Piezoelectric Materials

**Figure 4:**
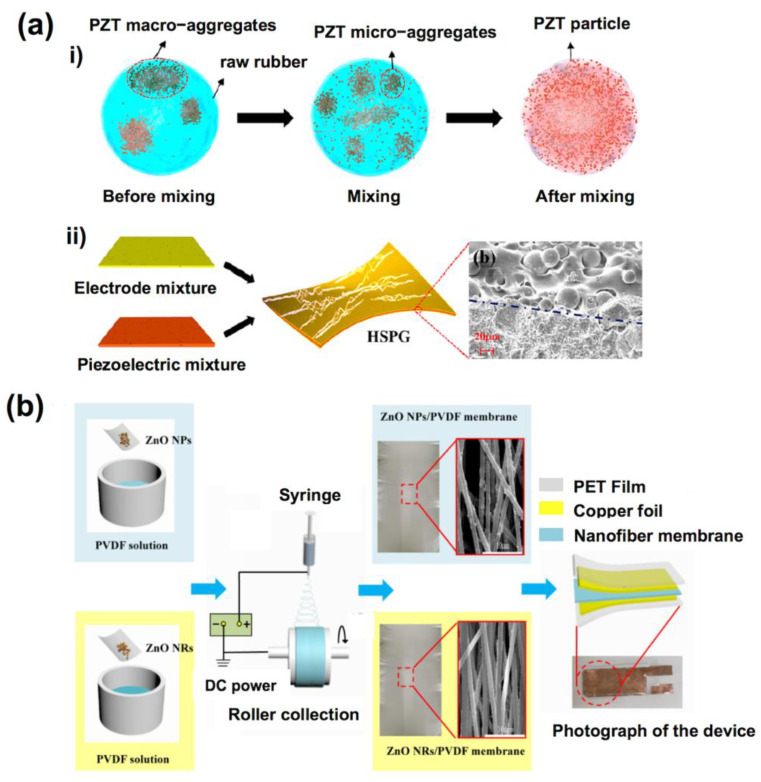
(**a**) Fabrication process and characterizations of the high performance PZT-based stretchable piezoelectric composite [61]; (**b**) schematic illustration of ZnO/PVDF nanocomposite fibrous membrane [62].

As mentioned above, piezoelectric polymers have good mechanical properties. However, their low piezoelectric coefficients limit the performance of sensors. Therefore, many typical inorganic piezoelectric materials, such as PZT [61] and ZnO [62], have been used to fabricate piezoelectric composites to improve the performance of piezoelectric polymer sensors. At the same time, the piezoelectric composites obtained by doping the inorganic fillers in the polymer also show better physical properties than the single component. Niu et al. prepared stretchable ceramic/polymer piezoelectric composite by mixing PZT with solid silicone rubber, with 92% filler mass ratio and 30% deformation (Figure 4a) [61]. In another work, Gu et al. prepared a novel flexible ZnO/PVDF nanocomposite fibrous membrane by electrospinning (Figure 4b) [62]. The study showed that the synergistic effect between the rod-like piezoelectric nanofillers and electroactive β-crystals of PVDF plays an important role in enhancing the piezoelectric behaviors of a ZnO/PVDF nanocomposite. 

## 4. Piezoelectric Sensors for Structural Health Monitoring

The following section presents an updated literature review of piezoelectric sensors for the structural health monitoring in the recent years. In order to elaborate clearly, different technologies are simply introduced and recent relevant progress on piezoelectric sensors are reviewed in detail. The piezoelectric SHM can act in both active and passive modes to realize the SHM. Active monitoring sensors can be utilized based on electro-mechanical impedance, guided wave propagation, or ultrasonic propagation, while passive monitoring sensors based on acoustic emission or stress wave propagation.

### 4.1. Sensors Based on Electro-Mechanical Impedance (Active Mode)

The electromechanical impedance-based (EMI) monitoring is one of the innovative and powerful structure health monitoring techniques. The electromechanical impedance-based SHM utilizes the electromechanical property of piezoelectric materials and the coupling of piezoelectric sensors and target structure. The EMI sensors can be attached to the surface of the structure or embedded into the structure, such as smart aggregates. In this active monitoring method, the piezoelectric EMI sensors can work as actuators, converting the electric voltage signal into a mechanical stress solicitation. Meanwhile, the piezoelectric sensors can act as the sensors, converting the structure’s mechanical response to an electric signal. The feedback electric signals can be further dealt, using algorithms to detect, localize, and characterize damages in the structure. The EMI methods are used for continuous monitoring and early detection of structural defects, such as joint looseness, debonding, and crack detection. Liang et al. were the first to propose the concept of the electromechanical impedance for the EMI system and suggested 1-DOF free-body diagram of a PZT–structure system to explain the interaction between the PZT and the host structure [63,64]. In addition, for the last decade, EMI sensors have been used to detect the occurrence, location, and characterization of damage in the concrete structures [65,66], composite structures [67], rotary blades in turbomachine [68,69], and bolted or adhesive joints [70,71]. Due to the limitation of damage-detecting area, EMI sensors are always attached to the positions that are important and prone to damage, such as bolted joints, adhesive joints, blades, etc. In the development of EMI sensors, the sensitivity and self-diagnosis of piezoelectric sensors have been investigated to guarantee the adequate application of EMI sensors.

**Figure 5:**
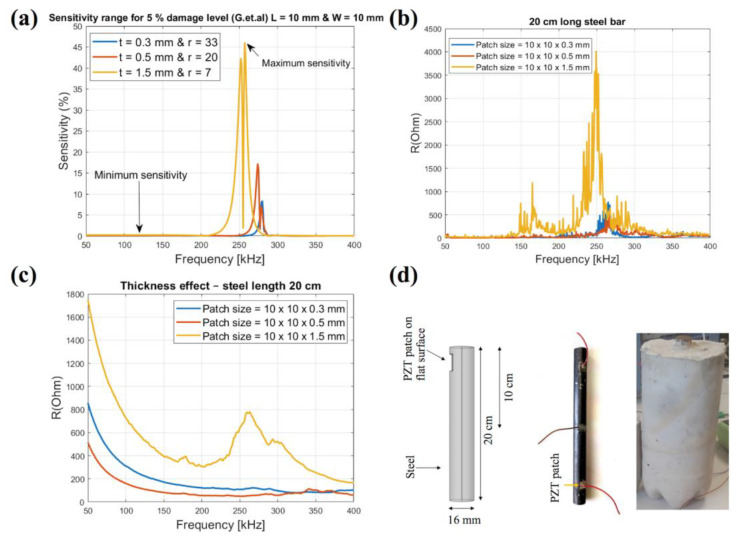
(**a**) Sensitivity levels with respect to thickness variations using Giurgiutiu’s model; (**b**) the real part of impedance in free air over frequency ranges from 50 to 400 kHz related to different patch size; (**c**) the real part of the impedance in reinforced concrete with varying patch thickness; (**d**) diagram of steel dimensions, actual steel, and actual concrete used in the experiments [74].

In order to ensure immunity to ambient noise and vibrations commonly present in practical applications, variable high frequencies (typically larger than 30 kHz) are preferred. This makes the EMI sensors sensitive to the minor damage; however, it also brings the issue of weak signals from far away, which limits the EMI sensor to the local damage monitoring. Some investigations on the design of EMI sensors have been conducted to improve the sensitivity of the monitoring system [72,73,74]. The sensitivity of detecting damage through EMI is closely related to the selected frequency band/wavelength of the excitation signal, which is emitted by the EMI sensor. The size of PZT piezoelectric sensor affects ZS/ZT (ratio of host structures’ mechanical impedance to the PZT transducer’s mechanical impedance), which is equivalent to the sensitivity. For using the frequencies below 125 kHz, the sizes (length and width) of sensors should fall into the range of 5 mm to 20 mm and thickness of sensors of 0.1 mm to 0.3 mm [72]. Hire et al. optimized the size of the piezoelectrical patch for optimum corrosion detection in reinforced concrete by combining theoretical and experimental studies [74]. They utilized the model developed by Giurgiutiu et al. to evaluate the impact of sensor patch sizes to the sensitivity range. They also measured the impedance of actual steel with sensor patch in air and in concrete. As shown in Figure 5, the experiment results are consistent with the theoretical results. In addition, it demonstrates that the appropriate design of the piezoelectric patch can improve its sensitivity for damage detection.

**Figure 6:**
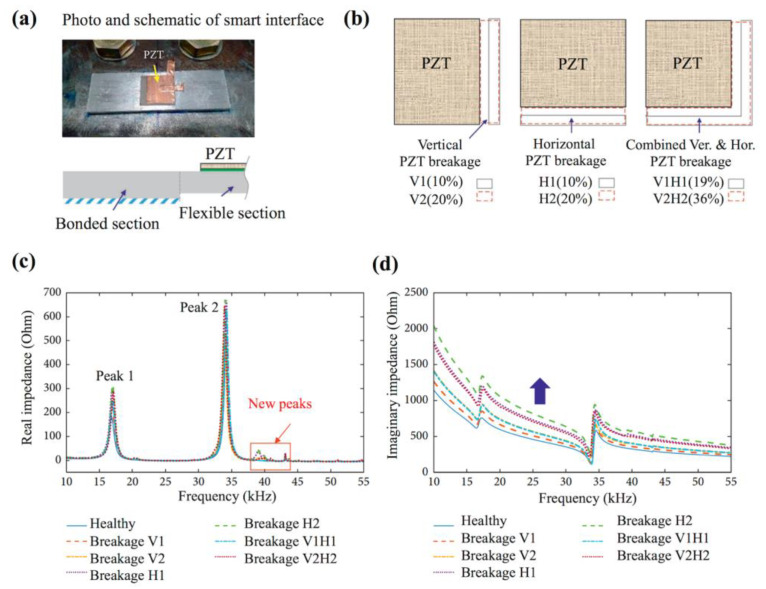
(**a**) Photo and schematic of smart interface with PZT transducer; (**b**) schematic of PZT breakage; (**c**,**d**) numerical EMI responses under PZT debonding defects, PZT breakage, and interface debonding defect [76].

Recently, the investigation of sensors with the capability of self-diagnosis for electromechanical health monitoring has become an essential issue [75,76,77,78]. Distinguishing sensor faults and functional degradations from structure damage will directly affect the effectiveness and accuracy of SHM. Jiang et al. introduced a K-means clustering analysis and artificial neural network to realize the self-diagnosis of piezoelectric active sensor for electromechanical impedance monitoring [75]. Three principal components, including the average change of conductance peak, the RMSD of susceptance, and the RMSD of conductance, were extracted by principal component analysis from the impedance signals. Then, the K-means algorithm was used to cluster different cases of sensor damages represented by the principal components. Finally, the analysis and artificial neural network were used to identify degree of the sensor damages. Four kinds of sensor damages, namely, pseudo-soldering, debonding, wear, and breakage, can be distinguished from the structure damage using the K-means clustering analysis based on admittance characteristics. Nguyen et al. established a finite element model corresponding to an experimental model based on a bolted steel girder connection to investigate the EMI response characteristics of a degraded piezoelectric-based smart interface [76]. Four common degradation types, including shear lag effect, transducer debonding, transducer breakage, and interface detaching, are simulated and their effects on EMI response are comprehensively analyzed. Figure 6 shows the photo and schematic picture of smart interface of PZT sensors. It was found that the transducer breakage occurring in the smart interface can result in unique shifts in the imaginary admittance and they can be feasibly diagnosed and differentiated from the structural damage through a diagnosis process, as shown in Figure 6.

### 4.2. Sensors Based on Guided Wave or Ultrasonics Propagation (Active Mode)

In this section, a typical structural health monitoring technique based on a specific wave, which can be guided wave or ultrasonics, is introduced. Guided wave, such as Lamb and Rayleigh waves, have the characteristics of surface propagation, low energy loss, and long propagation range. It determines that sensors based on guided wave propagation are technology of particular importance in SHM.

**Figure 7:**
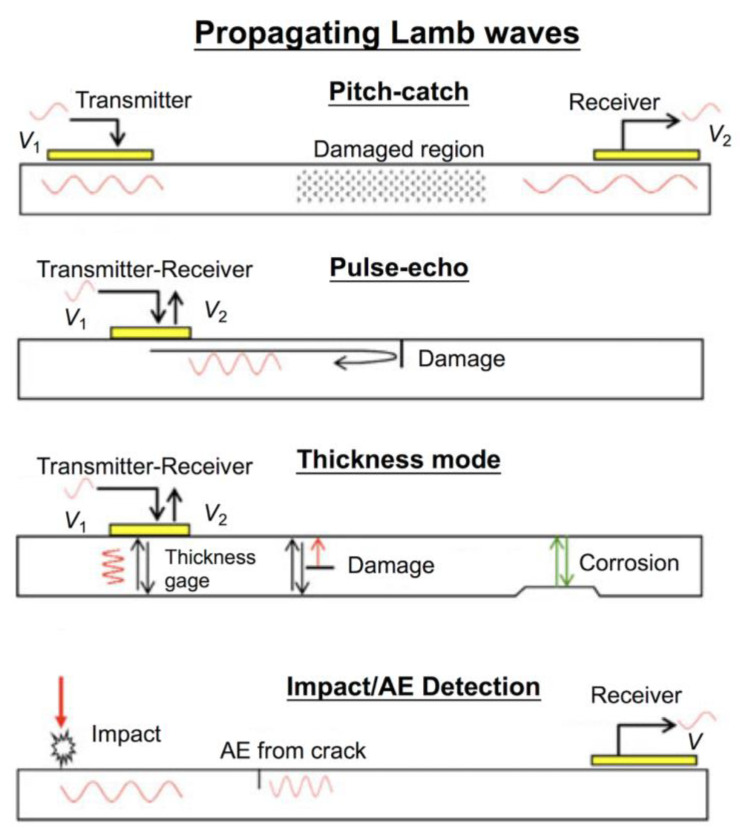
Modes of operation of piezoelectric transducers using propagating Lamb waves [79].

Guided wave-propagation-based SHM is most widely used for damage detection in metallic and composite structures, existing in aircraft, pressure vessels, missiles, pipelines, and steel bridges [14]. Piezoelectric components can be utilized as actuators or sensors in the guided wave-propagation-based SHM. According to the functionality of piezoelectric sensors, there are mainly four modes of the guided wave propagation, including pitch–catch mode, pulse–echo mode, thickness mode, and impact detection mode, as shown in Figure 7 [79]. Taking the typical pitch–catch mode as an example, a pair of piezoelectric transducers are attached on the plate-like structures. Firstly, ultrasonic guided waves are induced by the piezo-actuator attached to the surface of a flat plate-like structure. Secondly, ultrasonic disturbances occur and propagate radially to the around in the structure. Finally, the piezo-sensor around receives the electric charge signal, owing to the induced mechanical strains and output voltage signals (sensing waveform). While there is an even damage existing in the structure, the guided wave (such as Lamb wave) would incur dispersion and energy would attenuate during the propagation in the pitch–catch mode, pulse–echo mode, and thickness mode. The thickness mode can be used for the detection of corrosion thickness loss. In the impact detection mode, the piezoelectric sensors would receive a signal of acoustic guided wave, while impact events on the structure and advancing cracks occur. This is specific in acoustic emission, which will be introduced in detail in Section 4.3.

Compared with the local electromechanical impedance-based SHM, the guided wave monitoring technology can both realize local damage monitoring and global monitoring. In the case of local damage monitoring, the guided waves could be used to monitor the hybrid bonded joints. Jahanbin [80] utilized the ultrasonic interface guided waves that were propagating on the boundaries of bonded joints to inspect the disbond and delamination by recording the change in wave form, energy attenuation, and time of flight. For global monitoring, the selection or design of the piezoelectric sensors/transducers is of significant importance. To realize the global monitoring using a lower quantity of transducers, piezoelectric transducers exciting high purity of shear horizontal (SH0) wave are preferred. Boivin [81] optimized the geometry of the transducer to obtain a 23.0 dB SH0 wave using both simulations and experiments, and Zennaro [82] revised the transducer design (removed the wrap-around electrode) to eliminate the out-of-plane wave well in the propagation direction of fundamental SH0 mode. Ochoa et al. proposed a systematic multiparameter design methodology for the piezoelectric transducers used in SHM, and the multiparameter design includes the transducer shape, piezoelectric material, and transducer size [83].

**Figure 8:**
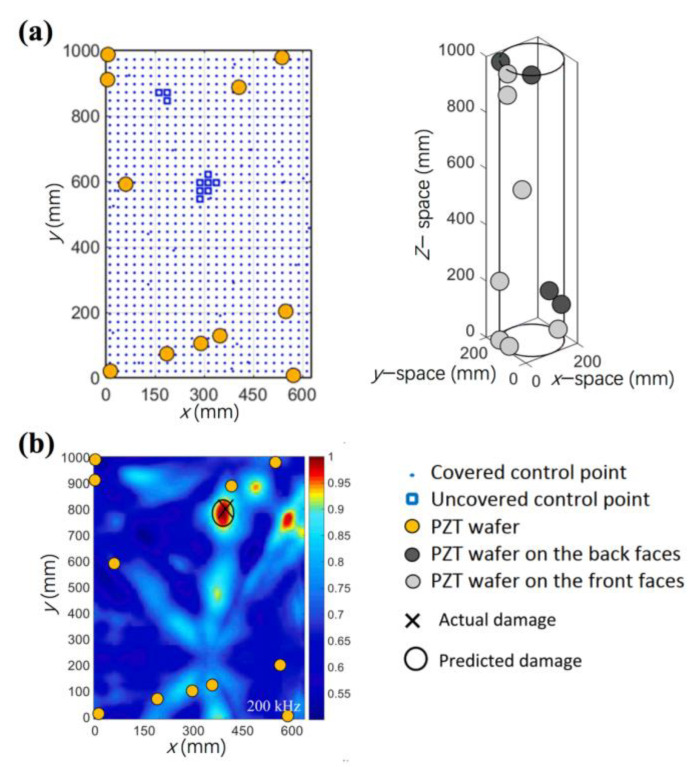
(**a**) A sensor network mounted on a pipe-like structure with 99% detecting coverage; (**b**) the location of damage identified on the pipe surface [86].

Apart from the transducers themselves, transducer/sensor network optimization offers an opportunity to obtain robust and low-cost active SHM systems. Two reviews had focused on the sensor networks for SHM by Ostachowicz [84] and Mustapha [85]. Sensor networks should be well-designed as they affect the SHM system integration, system performance, and accuracy of assessment. In the sensor network, deciding the number and locations of the sensors are the primary work for accurate damage detection and localization, then followed by the data transmission, data processing, etc. Many researchers have focused on determining the minimum number of piezoelectric transducers and achieving full or high coverage of damage monitoring by developing algorithms. A lot of algorithms, such as iterative optimization, combinatorial optimization, genetic algorithms (GA), and artificial neural network (ANN) techniques, have been developed to optimize distribution of sensors [85]. Recently, Ismail et al. proposed an approach of transforming any complex or closed structure surface, and then used a genetic algorithm to optimize the deployment of piezoelectric sensors [86]. The coverage of the optimized sensor network increases from 85 to 99% for a pipe-like structure, as shown in Figure 8a. Experimental validation was performed on a circular section (pipe) and the artificial damage can be accurately located within 18 mm from the actual location in Figure 8b. They also developed a similar model for sensor network optimization based on genetic algorithms, which was further validated on a large cargo door of an A330 airplane [87].

**Figure 9:**
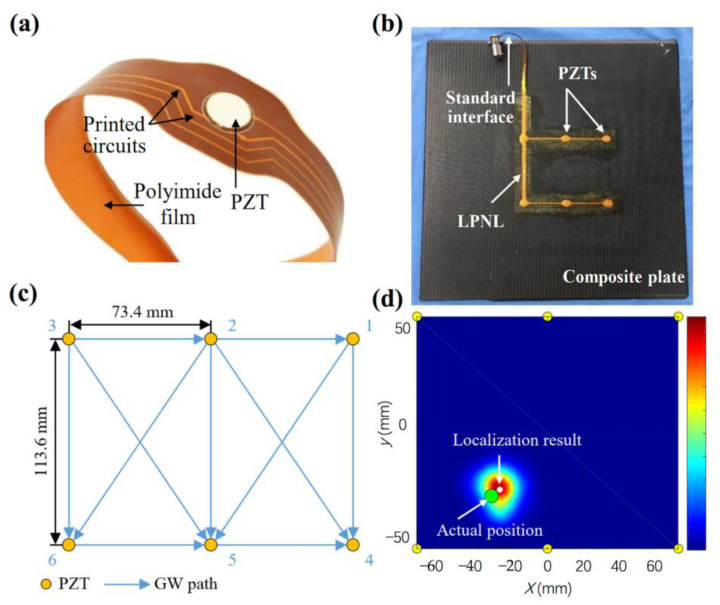
(**a**) Small section of LPNL and (**b**) the adopted LPNL, (**c**) its placement, (**d**) its imaging result of damage [91].

In addition to the optimization of sensor network for global monitoring, there are some interesting works on the topic of large area monitoring. Nonlinear lamb waves have attracted intensive attentions to be used for contact-type damages (e.g., disbands, delamination, and micro-crack) because of its high sensitivity to these damages [88,89]. Ju et al. proposed a nonlinear ultrasonic testing method for large-area monitoring of practical structures with arbitrary complexity using multi-mode guided waves [88]. When the multi-mode guided waves diffusely propagate through the structure, all available guided wave modes are automatically down-selected by the medium through attenuation or dispersion and the remaining modes efficiently transfer energy (for example, to their second harmonic modes) after encountering the micro-cracks. This method assures the success in detecting the crack that is close to the middle of actuator and sensor with a distance of 0.95 m. When large-scale monitoring is demanded, smart sensor networks are proposed to overcome the problems regarding large number of sensors, the complex cable, and placement efficiency. Many investigators developed the smart sensor networks combing the piezoelectric materials and flexible circuit technology [90,91,92]. The smart sensor networks have the advantages of light weight, high placement efficiency, and being suitable for complex structural forms. Ren et al. developed a large-scale PZT network layer (LPNL) design method based on FPC technology and one of the LPNLs connecting 37 PZT transducers has a dimension of 565 mm × 500 mm [91]. A small section of LPNL is shown in Figure 9a and one of the LPNL network is attached to the composite plate with the detail placement in Figure 9b,c. The maximum localization error of its damage diagnosis using this LPNL network is 0.41 cm, as shown in Figure 9d. 

### 4.3. Sensors Based on Acoustic Emission and Stress Wave (Passive Mode)

While the structures are subjected to impact by external force or irreversible damage with ultra-limited internal stress, the structures would generate transient elastic waves to release strain energy. Structure health monitoring based on this kind of elastic wave is also called acoustic-emission-based SHM. This typical acoustic-emission-based SHM is preferred to be introduced separately from the foregoing topics, as this method is a specific passive monitoring. Acoustic emission method is available only for damage initiation and propagation, such as impact event, crack initiation, fiber breakage, debonding, and delamination. For example, when a sudden crack occurs in the structure, piezoelectric sensors can catch the signal of acoustic emission from the crack. The acoustic-emission-based SHM is a local monitoring. Piezoelectric sensors should be placed near the key objects where damages or defects are prone to occurring.

**Figure 10:**
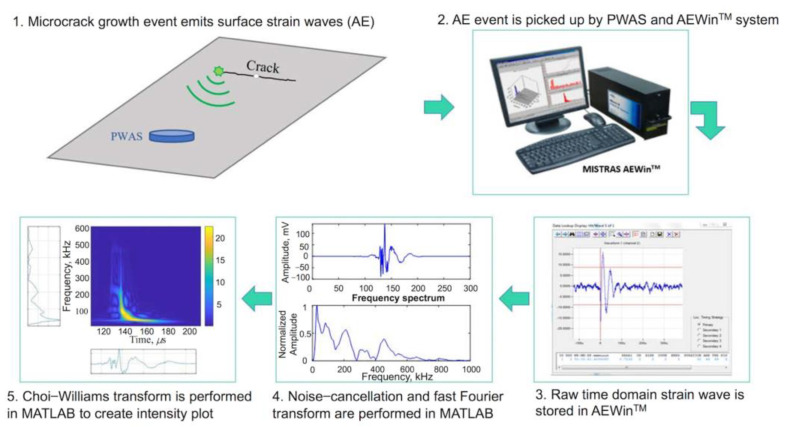
Flowchart of acoustic emission signal processing from microcrack growth event to the time–frequency intensity plot of Choi–Williams transform [101].

The acoustic-emission-based SHM has been deeply developed after its initial trail for damage monitoring. In the early research, the acoustic emission method was only used for the detection of the damage occurrence [93,94]. Then, this method was improved to localize the damage in metallic plates and composite structures [95,96,97]. Capineri et al. have reviewed the acoustic emission sensors and advanced methods for impact detection and localization [98]. Many papers have been published to improve the reliability and accuracy of detection and localization of damages, such as Akaike Information Criterion (AIC) for the accurate estimation of measured differential time of arrival [95], artificial neural network (ANN) [99], and theoretical modeling based on the phase velocity analysis [100]. In the recent years, much more advanced methods were evaluated to determine the type, magnitude, and severity of the impact or defects in the structures [101]. Garrett et al. proposed an artificial intelligence approach to estimate the fatigue crack length in thin metallic plates using acoustic-emission-based SHM [101]. Finite element modeling was firstly conducted to establish the simulated frequency spectra of calculated PWAS responses for different fatigue-crack-generated AE signals. Then, the Choi–Williams transform (CWT) result of the experimental inspected structure could be obtained from the raw acoustic emission signals following the process flow, as shown in Figure 10. It shows that a convolutional neural network (CNN) was successfully used for the artificial intelligence processing of the AE signals to predict the crack length. 

The piezoelectric stress/strain sensing technique is a promising approach of SHM techniques for dynamic loading to the structures, such as railway bridge [102] and aircraft wings [103]. When the structures are under low-frequency dynamic loadings, elastic waves would generate and propagate to the piezoelectric materials. The stress variation of structures can be reflected by analyzing the output voltage of piezoelectric sensors based on the direct piezoelectric effect. The application of piezoelectric stress/strain sensing technique for SHM has triggered worldwide interest after the first investigation by Krueger et al. [104]. Sha et al. developed an embedded smart piezoelectric sensor for concrete SHM [16]. The ratio of encapsulation materials is optimized to have proper mechanical performance. Furthermore, the mechanical sensing property of the embedded sensors with dynamic compressive loadings were studied in concrete, which demonstrates the great potentials of applying the piezoelectric sensors for concrete SHM.

### 4.4. Integrated Passive and Active Sensors

In some practical applications, only passive monitoring is not enough. For example, the impact events on airplane or aerospace structures might cause damage to the structures and the damage could also worsen or become extended with time due to the operational fatigue. In this scenario, the active monitoring is needed to continuously monitor the damage progression. In analogy, purely active monitoring also has its drawbacks. Piezoelectric transducers need to send out inspect waves continuously, resulting in useless work when they are not demanded. Therefore, it is desirable to integrate the passive and active sensors to overcome their drawbacks.

**Figure 11:**
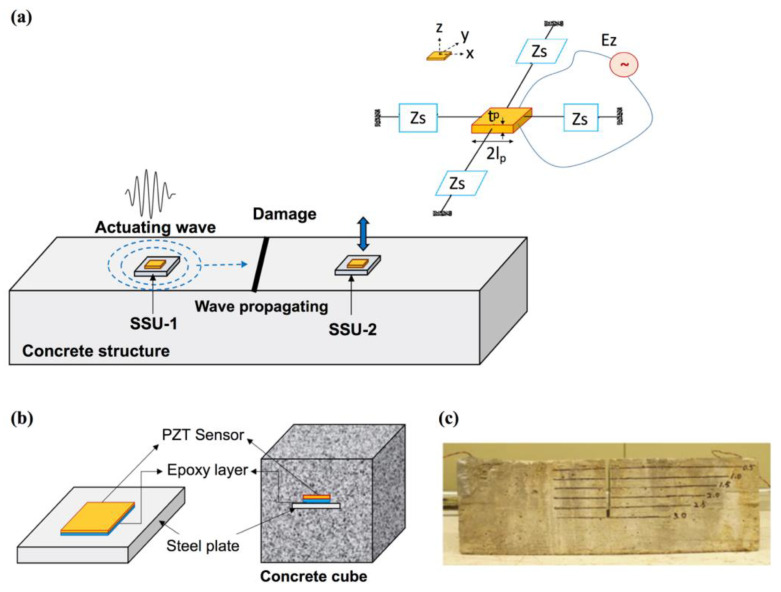
(**a**) Schematic representation of the combined EMI-WP technique for damage detection in concrete structure utilizing two SSUs; (**b**) the schematic diagram of concrete cube with embedded SSU and (**c**) an experimental specimen with embedded PZT patches and crack [107].

Bulletti et al. developed an integrated acoustic/ultrasonic SHM system for composite pressure vessels (massively used as fuel tanks) using the same piezoelectric transducers [105]. Two flexible arrays of PVDF interdigital transducers were designed. The transducers have two functionalities: passive detection of impacts and active damage assessment using guided Lamb waves. Guo et al. proposed a piezoelectric transducer-based integrated SHM system for impact monitoring and impedance measurement [106]. A “scheduling module” method is utilized to schedule the commands to the PZT sensors and transfer their signals to different preprocessing units for impact detecting or EMI measurement. This designed system is deployed in a supporting structure of a sailplane. Gayakwad et al. developed smart sensing units (SSU) to improve the effectiveness of the monitoring system, which contain PZT patches to detect both near-field and far-field damage in concrete structures through EMI and wave propagation techniques [107]. The PZT patch in the SSU-1 is used as an EMI admittance sensor for local damage identification and, meanwhile, the same EMI sensor is used to acquire elastic waves generated by another PZT patch in SSU-2 to monitor damages outside the EMI admittance sensor’s sensing area, as shown in Figure 11. Figure 11b,c show the schematic diagram of a concrete cube with embedded SSU and an experimental specimen with embedded PZT patches and crack, respectively.

## 5. Challenges, Opportunities, and Future Prospects

Piezoelectric sensors are indispensable for structural health monitoring. The last decades have witnessed many exciting developments of piezoelectric materials and sensors for structural health monitoring. Despite these breakthroughs, there is still plenty of room for further improvement, as exemplified by the following challenges.

Piezoelectric materials are the key component of piezoelectric sensors for structural health monitoring. The development of high-performance piezoelectric materials is essential to enable high-end piezoelectric sensors with exceptional sensitivity and further promote their practical applications in structural health monitoring. The combination of experimental synthesis, comprehensive characterization and the concept of materials genome, high-throughput calculations, and machine learning is expected to substantially accelerate the discovery of piezoelectric materials with unprecedented piezoelectrical properties [108]. As mentioned above, inorganic piezoelectric materials usually have good piezoelectric property but poor flexibility. In contrast, stretchable organic piezoelectric materials have low piezoelectric coefficients. Composite piezoelectric materials and sensors integrated with simultaneously high piezoelectricity and decent flexibility show unprecedented opportunity to improve the performance of SHM systems, such as working range, complex mechanical loadings, etc.

Piezoelectric materials always work as stacked wafers in the piezoelectric sensors and SHM systems, as shown in many reviewed examples. However, it increases the difficulty of placement and decreases the reliability, such as debonding fault. Piezoelectric films can be attached to the surface of the structure or embedded in the composite structure, which make them highly flexible and adaptable for deployment on complex configurations [92]. Advanced material processing technologies represented by additive manufacturing and three-dimensional printing offer new platforms to manufacture and implement piezoelectric sensors for structural health monitoring [109]. Piezoelectric materials, including piezoceramics, polymers, or composites, can be facilely processed into sensor components by these technologies.

So far, most of the work focuses on the research to address the deployment problems in SHM techniques in the category of experiment. From the experiment result to the practical applications, many problems need to be resolved, such as sensors self-detection, influence of environmental change on the monitoring signals, fundamental structure failure mode, and so on. For a reliable SHM system, especially in engineering system, accurate sensor self-diagnostics is one of the major issues. The sensor failures without self-identification will lead to false result in damage detection. For the long-term monitoring in the engineering SHM, sensor failure or debonding failure tends to occur. Different sensor faults need to be investigated to increase reliability of SHM systems [110]. Self-diagnostics for piezoelectric transducers have been investigated by much research based on the change in wave propagation signature or the change in EMI signature [77,111,112]. Sensor self-diagnostics would improve the reliability in the engineering SHM application in the future. The change in environment condition would bring error to the SHM systems. Lots of properties of piezoelectric materials would vary when the operating temperature changes, such as piezoelectric properties, dielectric constant, coupling constant, and Young’s modulus. For the guided-wave-based SHM systems, the temperature would make an impact on the guided wave baseline comparison and an optimal baseline selection method for the environment temperature range should be adopted [113,114]. For the EMI-based SHM technique, the dielectric constant exhibits the most significant effect on the electrical impedance of PZT sensor, which results in the shift of frequency and amplitude of impedance signatures [12]. Furthermore, the environmental effect of ambient induced noises, vibrations, and external loads also should be dealt with appropriately in the practical engineering SHM systems.

The material, sensor, and structural designs play an important role in the SHM systems. This phenomenon is particularly obvious in the EMI-based monitoring techniques. The working frequency range is decided not only by the design of piezoelectric sensor, but also by the structure properties. Traditional EMI methodology for indicating a failure is not enough. Developing a technique to determine the impact of damage on structural properties is demanding. Aabid et al. proposed some open research areas, such as packaging piezoelectric materials to generate a high working frequency range, integrating a network of sensors with local artificial intelligence (AI)/machine learning (ML) data-processing platforms and so on [10]. As reviewed above, only sensors based on guided wave propagation or acoustic emission have the opportunity to realize the global damage monitoring. However, structure health monitoring in the practical engineering applications is always preferred to cover the large area, such as in the bridge, aircraft, airplane, etc. It would make the SHM systems contain plenty of piezoelectric transducers and a complex line layout. The wireless sensor network to collect and process the information becomes the priority way to optimize the SHM system. Generally, bulky batteries are needed to provide power for the wireless sensor network, and these batteries should be frequently replaced due to the limited capacity. Therefore, a self-powered wireless sensor network is highly demanded, particularly for the case of structural health monitoring. The direct piezoelectric effect enables the piezoelectric devices to harvest electrical power from ambient mechanical and vibrational energies, such as structure vibration, airflow, etc. [115]. The piezoelectric energy harvesters can be integrated to the wireless sensor network for structural health monitoring to provide an unbounded power source for the system.

## 6. Conclusions

In this contribution, the recent progress in piezoelectric materials and sensors for structural health monitoring has been systematically reviewed. A brief introduction of the fundamental physical science of piezoelectric effect was introduced. Emphases are placed on the piezoelectric materials engineered by various strategies and the applications of piezoelectric sensors for structural health monitoring. Finally, challenges along with opportunities for future research and development of high-performance piezoelectric materials and sensors for structural health monitoring are highlighted. It is expected that the contribution could accelerate the development of high-end piezoelectric materials and sensors for structural health monitoring.

## Data Availability

Not applicable.
